# Heterotopic ossification of tendon and ligament

**DOI:** 10.1111/jcmm.15240

**Published:** 2020-04-15

**Authors:** Qiang Zhang, Dong Zhou, Haitao Wang, Jun Tan

**Affiliations:** ^1^ Department of Orthopaedic Surgery Shanghai East Hospital Tongji University School of Medicine Shanghai China; ^2^ Department of Orthopedics Changzhou No. 2 People’s Hospital Changzhou China; ^3^ Division of Geriatric Medicine & Gerontology Department of Internal Medicine Mayo Clinic Rochester MN USA; ^4^ Department of Physiology and Biomedical Engineering Mayo Clinic Rochester MN USA; ^5^ Department of Orthopedics Pinghu Second People’s Hospital Pinghu China

**Keywords:** heterotopic ossification, HOTL, ligament, review, tendon

## Abstract

Much of the similarities of the tissue characteristics, pathologies and mechanisms of heterotopic ossification (HO) formation are shared between HO of tendon and ligament (HOTL). Unmet need and no effective treatment has been developed for HOTL, primarily attributable to poor understanding of cellular and molecular mechanisms. HOTL forms via endochondral ossification, a common process of most kinds of HO. HOTL is a dynamic pathologic process that includes trauma/injury, inflammation, mesenchymal stromal cell (MSC) recruitment, chondrogenic differentiation and, finally, ossification. A variety of signal pathways involve HOTL with multiple roles in different stages of HO formation, and here in this review, we summarize the progress and provide an up‐to‐date understanding of HOTL.

## 1 BACKGROUND

Tendons and ligaments are similar in physiological functions, namely, mediate the attachments of the muscle to bone (tendon) and bone to bone (ligament) to transfer the strength and make the structures as a whole in the musculoskeletal system. They are also similar in histology, fibroblast‐like cells, hypocellularity and low vascularization. Tendons and ligaments are both filamentous collagen structures, of which the extracellular matrix (ECM) makes approximately 80% of the composition. The collagen is the main component of the ECM, which accounts for about 70‐80% of the dry weight, and the type I collagen accounts for 60‐85% of the total collagen. Tendons and ligaments start to form at approximately the same time in the mesodermal, which share many similar markers.

Heterotopic ossification (HO) of the tendon and ligament (HOTL) is common in the clinic, which is a poorly characterized degenerative disease with no effective treatment developed ever. Calcific tendinitis (CT), tendon ossification and ossification of the posterior longitudinal ligament (OPLL) are common HOTL diseases that disable the patients. Calcific tendinitis/tendon ossification is common in populations with high tendon injury risks like athletes and manual workers with repetitive tendon overuse, as well as in the elderly population, with severe pain and increased risk of rupture of the tendon.^1^ OPLL of the spine is a pathologic condition resulting in the narrowing of the spinal canal, causing various degrees of nerve/spine cord compression.^2^ As the similarities of the pathologies of heterotopic ossification in tendon and ligament (listed below), we discuss the representative type of HO, namely, HOTL here.

## 2 PATHOLOGICAL CHARACTERISTICS OF PRE‐HO STATUS IN TENDON AND LIGAMENT

Although several hypotheses have been put forward during the past decade, the pathophysiology of HOTL, in general, is still poorly characterized. But it is mostly believed that the HOTL is a failed injury repair process and usually begins with inflammation. Tendinitis, or tendinopathy, could be a vital status before the formation of tendon calcification. Similarly, the HO of the ligament has always been observed following either chronic or acute injuries. For example, OPLL cases, always saw in patients with cervical spondylosis, are resulted from repeated and chronic posterior longitudinal ligament injuries, as well as post‐surgery HO cases that result from acute ligament injuries.^3^

Histologically, tendinopathic tissue showed a non‐healing status characterized by hypercellularity, abundant proteoglycan deposition and collagen matrix degradation (as shown by matrix metalloproteinase 1 (MMP1) and tissue inhibitor of metalloproteinase 1 (TIMP‐1), and gelatinolytic activity). And much of the characteristics display similarity towards fibrocartilaginous metaplasia and calcium hydroxyapatite deposits like altered cell features and ECM composition.^4^ In patients with insertional tendinopathy and rotator cuff tendinopathy, the tendon samples display significant chondroid metaplasia, as shown by the acquired chondrocytic features of the tenocytes with the rounded appearance and a prominent nucleus, together with an up‐regulation of cartilage matrix and marker genes.^5^ Similarly, in a collagenase‐induced tendon injury model, chondrocyte‐like cells, as indicated by morphology and gene expression (type II&X collagen and Sox 9), suggesting that erroneous differentiation may reason for the failed tendon healing and subsequent ossification.^6^ In summary, excessive or inappropriate fibrocartilaginous matrix formation in tendons/ligaments may be considered as an essential process involved in developing tendinopathies and finally calcification.

### 2.1 Types of Heterotopic ossification

Heterotopic ossification commonly happens at all sites of the body, and there are two broad categories.^7^ 1. Non‐cell‐mediated HO, which is characterized by the direct deposition of calcium salts (ie dystrophic ossification, direct osseous metaplasia) without the help of osteoblasts or an osteoid matrix. 2. Cell‐mediated HO, which forms with the help of osteoblasts, via the process that produces an unmineralized bone matrix first and then mineralizes to mature and histologically normal bone. Furthermore, the cell‐mediated HO can also be classified into two kinds, intramembranous ossification and endochondral ossification. Intramembranous ossification, as mediated mainly by osteoblast, is an essential process for foetal development of the craniofacial skeletal system, where the bone is developed from the direct conversion of mesenchymal tissue, without a cartilage intermediate.^8^ A typical example of heterotopic intramembranous ossification is progressive osseous heteroplasia (POH), a rare genetic disorder caused by loss‐of‐function mutation of the gene GNAS.^9^ POH is the most severe kind of HO in which patients display severe extraskeletal ossifications even after minor trauma.^9^ The ectopic bone formation begins as early as infancy, and the patients usually die when they are young.

Endochondral ossification is also a natural development process of long bone as well as fracture healing and most of the acquired heterotopic ossification, which begins with the differentiation and hypertrophy of chondrocytes, and then replaced by osteoblasts. Fibrodysplasia ossificans progressive (FOP), a rare genetic disorder that caused by a gain‐of‐function mutation in the GS regulatory domain of the bone morphogenetic protein (BMP) type I receptor, ACVR1 (ALK2), is also formed via endochondral ossification.^10^

Then, which type of HO does the HOTL occur through? As both chondrocytes and osteocytes were found upon histological examination, and the appearance that chondroid metaplasia occurs around the ossification, it seemed that the intramembranous ossification alone was not possible to form the ectopic bone in HOTL. It is widely believed that HOTL occurs mostly via endochondral ossification, like the embryonic development of long bones.^11^, ^12^

In a model of subcutaneous implantation of growth plate chondrocytes, endochondral ossification occurs successfully, but not the articular chondrocytes.^13^ And similar results were found with the bone marrow mesenchymal stromal cells (BMSCs) if it has been treated with chondrogenic differentiation in vitro before, but fibrous tissue was formed if the chondrogenic differentiation of BMSCs is unfinished.^14^ Moreover, subcutaneous implantation of osteochondral tissues is also able to recapitulate the endochondral ossification process that occurs during natural skeletal system development.^15^ Consequently, it seems the endochondral ossification is a natural process that happens in vivo*.* Is it most essential to form chondrocytes during endochondral heterotopic ossification, as the following process is just to leave the nature play its role? No direct evidence has been obtained. A previous study demonstrated that pathologic ectopic bone after trauma was limited in the presence of diabetes, which may be explained by the impaired early Sox9 activation (chondrogenic differentiation) and late bone resorption by osteoclasts caused by diabetes.^16^ Another paper also provided indirect evidence. When mature HO with limited aggression formed, no cartilage could be detected; however, when the HO reoccurred after excision, the renewed cartilage also formed as shown with Safranin O staining.^17^

Moreover, as the HO is thought induced by the error differentiation of the progenitor cells, the multi‐potential stem cells were not differentiated into tenocytes/ligament cells but others. As it is known, the tendon and cartilage share many similarities. The tendon morphogenesis is strongly associated with chondrogenesis during embryonic development. Also, although scleraxis is typically considered as a tendon marker, it plays an essential regulator of gene expression in chondrogenesis.^18^ And a unique kind of progenitor cells (Sox9+ and Scx+) has been proved to contribute to the establishment of the tenon‐to‐bone junction.^19^ Consequently, as the close relationship between tenocytes and chondrocytes, it seems more natural for the progenitor cells to error differentiate into the chondrocytes but not the osteocytes.

Interestingly, in ample researches on the reason for HO of HOTL, especially in OPLL, the osteogenesis differentiation is considered as the evaluation criterion and research object but not the chondrogenesis differentiation. The osteogenesis differentiation is just a natural process following chondroid metaplasia during endochondral ossification, and the induction of osteogenesis differentiation in animal models cannot reflect the exact situation of HO via endochondral ossification at all.

## 3 THE AETIOLOGY OF HOTL

**Figure 1:**
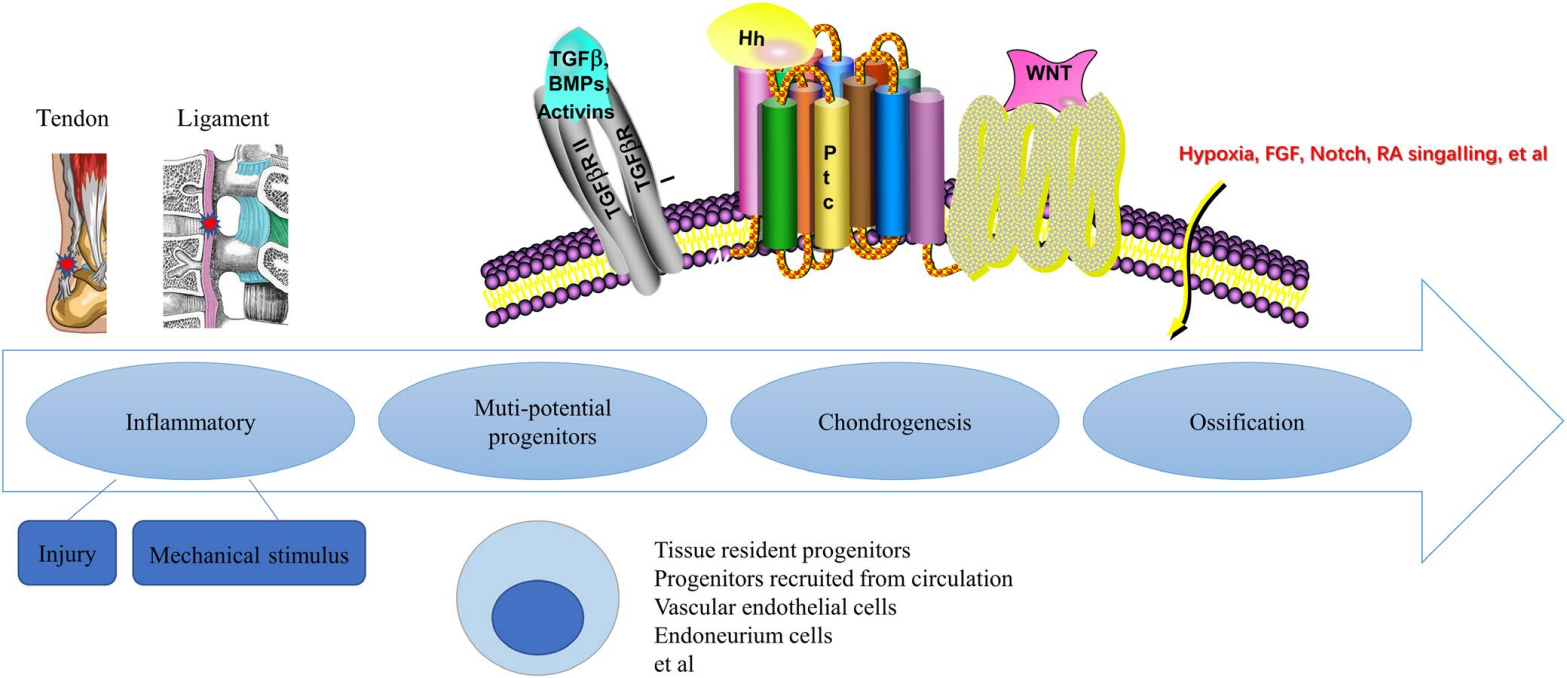
The summary of the mechanisms of HOTL development

The aetiology of HOTL is generally considered as a tissue repair process gone away although the mechanism is still far from understood. The process of HO formation is similar to the fracture repair,^20^ which is a highly complex and arranged physiological process including haematoma formation, tissue inflammation, MSC recruitment, skeletal tissue regeneration, extracellular bone matrix accumulation, angiogenesis and bone remodelling. And similarly, the process of HOTL includes the trauma/injury, inflammation, mesenchymal stromal cell (MSC) recruitment, chondrogenic differentiation and ossification formation (Figure 1).

### 3.1 Mechanical stimulus

Mechanical factors include overload, and repeating motions may be an essential factor in HOTL pathologies, as proved by the high morbidity of HOTL in athletes. Tendon and ligament, with the function of translating forces, are easy to be affected during daily life, and the wrong way of the following tissue repair process is considered to be one of the origins of HO formation. For example, thoracic ossification of ligament flavum (TOLF) is mostly found in the lower thoracic spine, which is a mobile transition region with a high risk of injury that experiences high tensile forces, mechanical overload and repetitive tensile strain.^21^

Besides the role of mechanical stimulus in inducing injury, it was reported able to induce chondrogenic/osteogenic differentiation, which may be an essential factor for HO formation.^22^ In vivo, repetitive loading and repeated compression were able to induce fibrocartilage phenotype by expressing more cartilage genes.^23^ In vitro, tendon stem cells and MSCs can be induced to differentiate into the chondrogenic/osteogenic lineages following a mechanical stimulus, as proved by elevated expression levels of chondrogenic/osteogenic markers.^24^, ^25^

### 3.2 Injury and inflammatory

Despite the lack of infiltration of inflammatory cells in tendinopathic tissues/OPLL, the inflammatory response may still be closely associated with the development of HOTL. The local inflammation levels and serum concentration of hs‐CRP (high‐sensitivity C‐reactive protein) in the OPLL samples were much higher in comparison with the normal posterior longitudinal ligament tissues. Moreover, a weak positive correlation was also observed between the average increase in the OPLL length per year and the serum concentration of hs‐CRP.^23^ Similar results were found in other types of HO, like HO after total hip arthroplasty (THA) and traumatic spinal cord injury.^26^, ^27^ In hereditary cases, ectopic ossification always occurs in response to minor injuries and, subsequently, local inflammation ^28^; in acquired HO cases like traumatic HO, the extent and severity of ectopic ossification are closely correlated with the degree of injury and inflammatory incurred.^29^ And more importantly, the non‐steroidal anti‐inflammatory drugs (NSAIDs) are useful in preventing HO.^30^ A previous isobaric tags for relative and absolute quantification iTRAQ analysis also confirmed the role of inflammation response in the mechanisms of HOTL and reported a list of inflammation‐related factors include tumour necrosis factor‐α (TNF‐α), insulin‐like growth factor II (IGF‐II), insulin‐like growth factor‐binding protein 5 (IGFBP5), prostaglandin reductase 1 (Ptgr1), latent‐transforming growth factor beta‐binding protein 3 (LTBP3), transforming growth factor beta‐1 (TGF‐β1), neutrophil elastase (NE), serum amyloid A‐4 protein (SAA4), protein S100‐A9 and prostaglandin‐H2 D‐isomerase (PTGDS).^31^ In vitro, TNF‐α was able to induce the expressions of Osx and BMP2 in ligamentum flavum cells in a dose‐dependent manner, as well as the osteogenesis related genes OCN and ALP. And the potential mechanism of TNF‐α in TOLF probably depends on its function on regulating cell proliferation and promoting osteoblast differentiation.^32^ BMP and TGF‐β are also shown abundant in the ossification areas, together with the adjacent cartilaginous areas of OPLL.^33^

In summary, local inflammation may be an important origin of HOTL pathologies, similar to the results found in other types of HO and bone remodelling processes.

## 4 THE MECHANISM OF HOTL

### 4.1 Cell lineages contributing to HO formation

Many hypotheses and efforts have been put forward to figure out the cells responsible for HO formation, and the erroneous differentiation of stem cells is believed to be responsible for it (Figure 1). MSCs were recruited to the injury site, and the environmental niche that consisted of other cell types and microenvironmental factors are critical roles to influence the fate of MSCs. Consequently, dysregulation of key microenvironmental factors may cause loss‐of‐function or gain‐of‐function changes of MSCs, which leads to deficiencies in tissue regeneration or leads to ectopic bone formation. Till now, various types of progenitors have been detected as the origin of HO, including tissue‐resident progenitor cells, mesenchyme stem cells recruited from the circulation, vascular endothelial cells, endoneurium cells and et al.^34^, ^35^, ^36^ For HOTL, the tissue‐resident progenitor cells were mostly researched.

### 4.2 Tissue‐resident progenitor cells

The tendon and ligament tissue contain a unique cell population, the so‐called tendon‐derived stem cells (TDSCs)/ligament‐derived stem cells (LDSCs), demonstrated clonogenicity, self‐renewal and multi‐differentiation potential, which is one of the possible sources of progenitors contributes to HOTL. Yu PB and Levi B demonstrated that the connective tissue‐resident progenitor cells, as characterized by tendon marker Scx+, contributed to the initiation and progression of HO in tendon and ligament.^34^, ^35^ Also, bone‐chondro‐stromal progenitor (BCSP) cells (AlphaV+/CD105+/Tie2‐/CD45‐/Thy1‐/6C3‐) were demonstrated involved in the pathologies of HO, although not contributing mainly to HO formation.^37^ The CD105 negative subpopulation of the tendon progenitor cells was found able to induce chondrogenic degeneration in injured tendons.^38^

### 4.3 Other cell resources

Several studies have specified that the progenitor of HO is a vascular endothelial cell, a multipotent progenitor resident in the tissue,^39^, ^40^, ^41^ as shown by lineage tracing studies of Tie2+ cells. The endothelial cells were reverted to a mesenchymal cell phenotype via the endothelial‐to‐mesenchymal transition process (EndMT), allowing them to migrate into the injury site and then undergo re‐differentiation. However, another study indicated that the EndMT, although present, was mot a major cell resource of trauma‐induced HO.^42^ Other types of cells like endoneurium or neural cell were also reported to be candidate cell sources of HO,^36^ but no such researches have been performed on tendon or ligament.

An explanation of the reason why so many different types of origin were detected is the tracking technique present was not specific, and the progenitor cells at different time‐points may express different markers. For example, Tie2+ is widely used to define the endothelial cells. However, it has also been proved to exist in various cell types, including inflammatory cells, chondrocytes and progenitor cells.^43^ Whether the different lineages mark the same mesenchymal cell type in different tissues or represent distinct progenitors need to be explored. It would also be exciting and valuable to figure out whether these progenitor cells differentiate into the cartilage and bone lineages directly or through a reprogramming process into mesenchyme stem cell intermediate.

In summary, in HO cases, immanent or recruited MSCs caused by local injury and subsequently inflammatory, home, or migrate to the injury site and then give rise to osteo‐chondrogenic differentiation in reply to a chain of complex cell signalling cascades including various growth factors and inflammatory cytokines.

### 4.4 The microenvironment influencing cell fate

Multiple signalling pathways seem to be implicated in the pathogenesis of HO. Recent data have suggested the probability of the central role of the BMP pathway in HO pathogenesis, along with other signalling such as TGFβ, Hedgehog (Hh), Wnt/β‐catenin, HIF‐1α and et al (Figure 1).

#### 4.4.1 Bmp signalling pathway

The predominant role of BMP signalling in HO has been supported by numerous experiments either on hereditary or acquired HO.^44^, ^45^, ^46^ BMPs, as one of the classical osteoinductive growth factors, belong to the TGF‐β superfamily, which bind to the type I subunits receptors and finally mediates the signalling through Smad 1/5/8 dependent or independent mechanisms. BMP signalling plays critical roles in natural skeletal system development, including inducing proliferation and differentiation of condensing MSCs towards chondrocytes and osteocytes.^47^ The mouse line that overexpresses BMP4, as well as the ectopic injection of BMP 2 and 4, is widely used as models of HO.^48^ BMP‐4/7 is reported to be involved in regulating late events in tendon ossification.^49^ It has also been reported that BMP and its receptors are widely expressed in the ossified and adjacent chondrogenic areas of OPLL and thoracic ossification of the ligamentum flavum (TOLF).^33^, ^50^ In FOP, the GOF mutation of the type I BMP receptor ACVR1 introduces ectopic bone formation after minimal soft tissue trauma.^51^ Similarly, two novel variants of the gene BMP‐2 have also been proved to induce TOLF in Chinese Han population.^52^ BMP‐2 and BMP‐7 are also able to induce local inflammation, which has been proved important in the pathology of HO happening.^53^

#### 4.4.2 TGFβ signalling pathway

The role of TGFβ signalling has also been widely reported in all kinds of HO. And importantly, as the function of TGF‐β superfamily proteins in regulating the balance between the chondrogenic and tenogenic transcription factors Sox9 and Scx, the role of TGFβ in HOTL has got much more attention than other types of HO.^19^, ^54^ It is also interesting that based on different cell types, different experiment environments or even different time‐points, the TGF‐β has been shown to have both the abilities of chondrogenesis and tenogenesis. In vivo application of exogenous TGFbs to the interdigital embryonic limb also induces ectopic cartilages ^55^; however, similar treatments in early limb mesenchyme exert an anti‐chondrogenic influence.

In vivo, it was reported that TGF‐β was present in the ossified and chondrogenic areas of OPLL.^33^ Moreover, in trauma‐induced HO of Achilles tendon model, TGF‐β was shown able to initiate and promote HO in all stages, whereas systemic injection of a TGF‐β neutralizing antibody was able to attenuate the ectopic bone formation.^11^ Activin A, another member of the TGF‐β superfamily, was also found able to trigger HO in FOP.^56^ In vitro, some studies reported additional TGFβ increases chondrogenesis of limb mesenchymal cells cultured at high density,^57^ as well as other cell lineages, including bone marrow‐derived stem cells.^58^ However, another study demonstrated TGF‐β ligands down‐regulate Sox9 and up‐regulate Scx and tenomodulin expression in micro mass cultures of MSCs.^54^ Moreover, the chondrogenic differentiation effect of high‐density mesenchymal cell cultures can be reverted to fibrogenic when TGFβs are added.^59^, ^60^ The mechanism of how TGFβ signalling induces HO formation remains unknown, and hypotheses like inflammation promotion, MSCs recruitment, chondrogenic differentiation and the ossification formation have all been widely researched, but no conclusion has been got yet.

#### 4.4.3 Hedgehog signalling pathway

Hedgehog (Hh) signalling is important for natural skeletal development, both for chondrocyte differentiation and the formation of osteoblasts. Hh signalling alone, or together with various other factors like BMP signalling, was also reported both in fracture healing and heterotopic bone formation.^61^

In POH cases, the Hh signalling was found activated in osteoblasts and progenitor cells.^62^ Similarly, in endochondral ossification cases like OPLL and BMP2‐induced HO, the over‐activation of Indian Hh (Ihh) signalling can also induce excessive chondrogenesis and finally ectopic bone formation,^12^, ^63^ especially in pre‐hypertrophic and hypertrophic chondrocytes during the early stages.^64^

The mechanism of how Hh pathway participates in HO progression remains uncertain; further researches are required to discover whether or not the Hh signalling is also essential for late‐stage tendon ossifications, as well as how Hh signalling is working.^64^

#### 4.4.4 Wnt/β‐catenin signalling pathway

The canonical Wnt/β‐catenin signalling is essential and widely expressed in various tissues during development, and it appears particularly important for bone biology,^65^ which functions as mediators of Hh and BMP signalling.^66^ For example, the Wnt pathway works together with the BMP signalling and contributes to chondrocyte hypertrophy via the regulation of Wnt signalling receptor lipoprotein receptor‐related protein 5 (LRP‐5).^67^ Moreover, Wnt/β‐catenin signalling is also found able to promote BMP signalling by increasing the expression of BMP2.^68^

Several studies have also linked Wnt signalling with both hereditary and acquired HO. In POH cases, Wnt signalling cooperates with the Hh signalling to induce the osteogenesis.^62^ Increased expression of Wnt signalling was observed in post‐trauma HO cases.^69^ Another study further demonstrated the aberrant Wnt signalling contributed to HO formation in a patient with adrenal myelolipoma.^70^ The relationship between Wnt pathway and HOTL was mostly discussed in ligament ossification cases. Lots of researches demonstrated the OPLL was mediated by aberrant Wnt signalling, and the ossification area of the spinal ligament correlated with the Wnt signalling pathway activity.^71^, ^72^ Also, Wnt/β signalling was shown able to mediate osteogenic differentiation induced by mechanical loading in TDSCs.^73^

#### 4.4.5 Hypoxic Cell Signalling pathway

Hypoxia‐inducible factor 1‐alpha (Hif1‐α), known as a crucial transcriptional regulator in responding to hypoxia, has been intensely associated with lots of natural develop processes and diseases, and no exception with HO. The hypoxic environment stimulates chondrogenic differentiation of progenitor cells, which is widely proved in the development of skeletal system.^74^ In a rat Achilles tendon ossification model as well as in an FOP mouse model, HIF‐1α was significantly up‐regulated during chondrogenic differentiation stage.^75^ The effect of cellular hypoxia on inducing HO was based on amplifying BMP signalling.^76^ Further studies reported that Hif1‐α was critical for HO in immature stages, and several inhibitors have been developed to prevent and treat HO.^77^

#### 4.4.6 Other signalling pathways

Several other signalling pathways are also involved in HOTL process, including FGF, Notch, Retinoic acid signalling and et al, which contribute unequally at different stages of HO. For instance, as injury usually triggers the process of HO, the injury response and inflammatory signalling pathways are crucial at the early stage. Then, MSCs recruitment and differentiation signalling pathways are dominant, including hypoxia, FGF, integrins and the TGF‐β/BMP signalling cascade. While at the later stages, osteogenic signalling pathways play key roles. In a word, various pathways contribute to the same goal with different roles at different stages of HO.

## 5 TREATMENT OF HOTL

**Figure 2:**
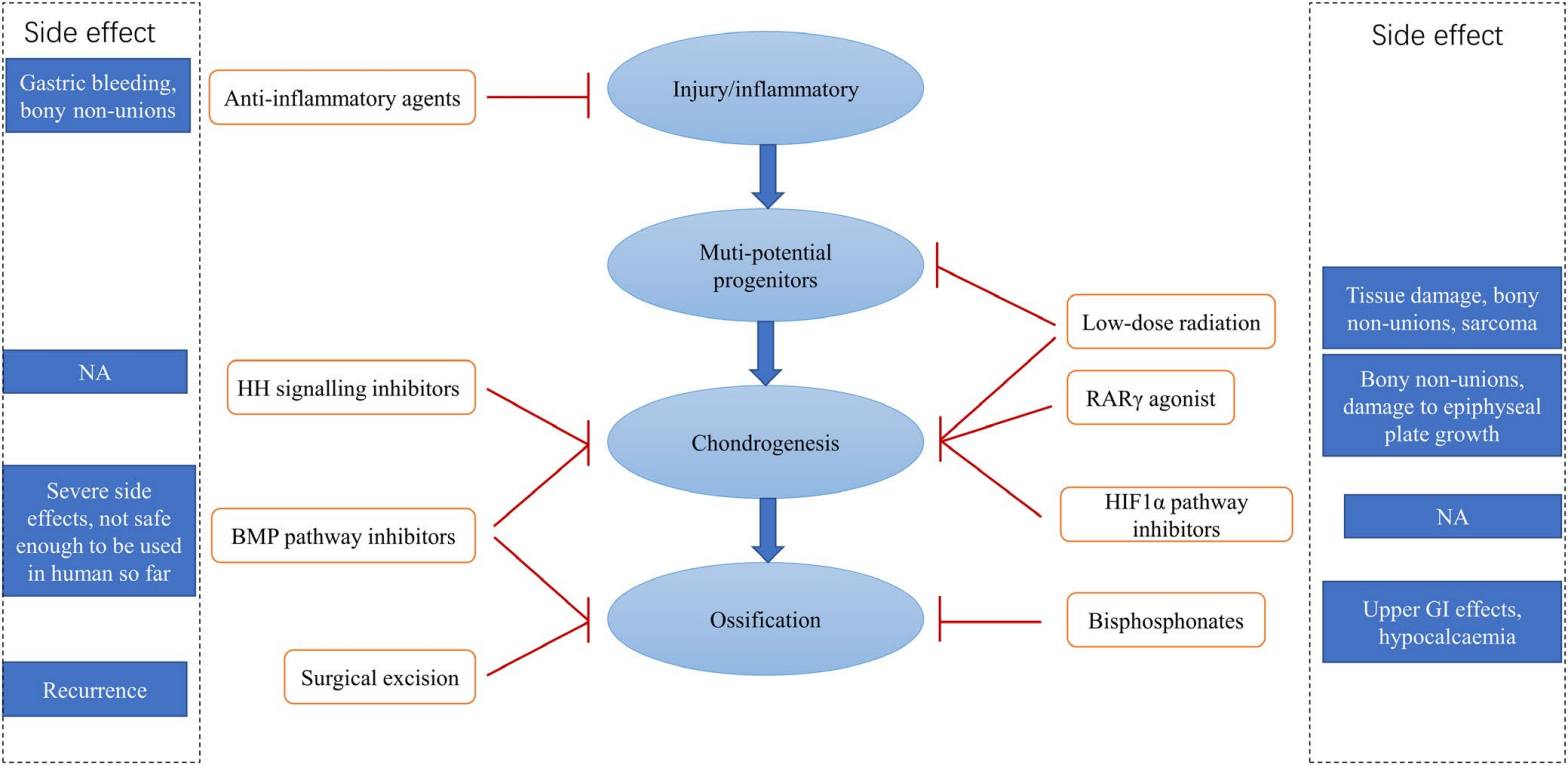
The summary of treatments for HO

Because of the poor understanding of the pathological mechanism, no optimal treatment strategies have been developed until now. Surgical excision is the most useful and preferred method for clinical application of present ossifications. However, the risk of recurrent post‐surgery heterotopic ossification could not be ignored ^3^ due to the high susceptibility of ossification for these patients. Also, as for spinal ligament ossification cases, nearly all ossifications could not be removed because of the limited surgical space between the ossification and spinal cord. Thus, the remaining ossification area continues growing, and some of the cases progress to a second surgery.^78^ Consequently, more and more attention has been put on other treatments based on the current findings of molecular and cellular mechanisms (Figure 2).

### 5.1 Low‐dose radiation

Low‐dose radiation has been widely used in preventing post‐surgery or recurrent HO. The radiation damage to the natural tissue could not be avoided, however, as the disproportional effect of it on HO progenitors and resident cells, the radiation treatment still benefits a lot for high‐risk HO cases, regardless of side effects like bony non‐unions or radiation‐induced sarcoma.^79^ The use of radiation in preventing HOTL is rarely researched, possibly because of the side effects associated with its use, especially for spinal ligament ossification cases that may result in serious spine cord injuries.

### 5.2 Anti‐inflammatory agents

Considering the important role of inflammatory in initiating HO, the use of anti‐inflammatory agents [(corticosteroids or non‐steroidal anti‐inflammatory drugs (NSAIDs)] has been widely used for any kind of HO.^30^ The NSAIDs were commonly used in OPLL and ossified tendinitis cases to release pain and prevent the progression of ossification.^30^, ^80^ Besides, recent researches have also demonstrated more mechanisms of anti‐inflammatory agents in treating HO like differentiation regulating ability.^81^ For example, cyclooxygenase‐2 inhibitors work via the suppression of prostaglandin and thus reducing inflammatory reaction. Besides the function of prostaglandin in mediating inflammatory reaction, it is shown necessary for osteogenic differentiation of MSCs during bone growth and healing.^82^, ^83^

### 5.3 BMP pathway inhibitors

Considering the critical role of BMP signalling in HO developing, the use of BMP pathway inhibitors has got lots of attention in the past decade to mitigate ectopic bone formation.^84^, ^85^ Small molecule inhibitors like dorsomorphin and LDN‐193189 have been developed to block the ACVR1/ALK2 signalling to reduce HO in FOP.^84^, ^85^ However, as the important role of BMP signalling in almost every element of life activity, the cytotoxicity and side effects could not be ignored and thus limits the application of it other than genetic cases. Besides the selective BMP signalling inhibitors, other drugs with the ability to inhibit BMP activity were also used. In an injury‐induced HO model of mouse, the application of apyrase, which is able to inhibit the SMAD1/5/8 phosphorylation indirectly, was found useful to reduce the HO formation after burn injury and tenotomy.^86^

### 5.4 RARγ agonist

Since chondrogenesis requires a decrease in retinoic acid receptor (RAR) expression, the use of a RAR agonist is considered useful to reduce chondrogenesis and thus heterotopic endochondral ossification.^87^ NRX204647, a selective RAR‐γ agonist, was found able to eradicate HO formation in mice by preventing differentiation of MSCs into chondrocytes.^87^ Similarly, in a rat blast injury model, the progression of blast injury‐related HO was inhibited by treating with the RAR γ agonist, Palovarotene.^88^ More importantly, the RAR‐γ agonists seem safer than the BMP inhibitors with less adverse and side effects, as shown in the phase 2 clinical trial of palovarotene.^89^

### 5.5 Hh signalling inhibitors

Given the importance of Gαs and HH signalling in POH, the use of HH signalling inhibitor has also been widely researched.^90^ Drugs such as arsenic trioxide (ATO) and GANT58 have been found to reduce HO in the POH models.^91^ However, no researches based on other types of HO have been done yet.

### 5.6 HIF1α pathway inhibitors

Considering the important role of hypoxic signalling in inducing chondrogenesis and initiating inflammation, HIF1α inhibitors are also developed for HO prevention. In recent research, the HIF1α inhibitor PX‐478 or rapamycin were both found useful in preventing either genetic or burn/tenotomy‐induced HO.^77^ Besides, the hydroxyethyl starch (HES), which enhanced the microcirculation and thus interrupted the hypoxic microenvironment, was found able to inhibit HO progression too.^92^ Similar results were found in a post‐spinal cord injury HO model base on the pulse low‐intensity electromagnetic field (PLIMF) therapy, which functions via increasing local blood flow.^93^

### 5.7 Bisphosphonates

Although previously mentioned drugs are all potential for HO prevention, none of them can completely prevent HO, as well as no drug is useful for existing HO. However, the bisphosphonates, like disodium etidronate, are considered effective for both preventing and treating the existing HO,^93^ as proved in various HO types including neurological ^93^, ^94^ and burn injury‐induced HO.^95^ The mechanism how bisphosphonates reduce HO remains uncertain. Bisphosphonate is commonly considered as anti‐resorptive agent, and thus, the effect of it in treating HO seems contradictory. One explanation is the bisphosphonates may non‐selectively affect both osteoblasts and osteoclasts, and thus reduces osteoblasts and HO formation.

## 6 CONCLUSION

Similar to other kinds of HO, the HO of tendon and ligament forms via endochondral ossification. Because of the similarities of the tissue characteristics and pathologies of the tendon and ligament, the mechanism of HO formation of tendon and ligament was discussed together here. The HO procedure is a dynamic pathologic process that includes trauma/injury, inflammation, MSC recruitment, chondrogenic differentiation and, finally, the ossification formation. A variety of different conserved signal transduction pathways were involved in different stages of ectopic bone formation, and till now, no effective treatment has been developed.

## CONFLICT OF INTEREST

The authors declare that they have no conflict of interest.

## AUTHORS' CONTRIBUTIONS

Qiang Zhang prepared the manuscript. Dong zhou, Haitao Wang and Jun Tan conceived the idea for the workshop and helped to improve the quality of the manuscript. Qiang Zhang, Dong Zhou and Jun Tan were granted funding. The authors researched, discussed and approved the concept, drafted and submitted the commissioned paper. All co‐authors made a significant intellectual contribution to the concept of the manuscript.
